# Knowledge, attitude and practice of safe sex among men who have sex with men (MSM) in Nigeria

**DOI:** 10.1186/1742-4690-9-S2-P234

**Published:** 2012-09-13

**Authors:** RG Ezomoh

**Affiliations:** 1 The Initiative for Equal Rights, Lagos, Nigeria

## Background

The success of the antiretroviral drugs in the late 90s resulted in increase sexual risk-taking behaviors among MSM and “business as usual” attitude to sex which has resulted in the high prevalence of HIV infection. We therefore studied the knowledge, attitude and practice of safe sexual practices among men who have sex with men (MSM).

## Methods

Self administered open ended questionnaire with four sections: Biodata, knowledge, Attitude and practice of safe sexual practices were administered. 8 MSM communities were selected using stratified sampling method and a key opinion leader was selected per community; the KOLs administered the questionnaire to a total of 1600 MSM (200) per community.

## Results

1600 participants were recruited into this survey and all were MSM (100%). 17% were aged 18-20years, 65% were between the age 21-29years while 10% were aged 30-40 years and 8% were over 40years of age. 96% of our respondents had good knowledge of condom use while 39% were informed about the advantage of lubricants for anal sex. 67% admitted to inconsistent use of condom. All our participants have been involved in 3 casual relationships in the last 12months. 78% had unprotected anal sex in the last 3 months with and 88% of them were between the 18-29 years of age. 64% had oral sex in the last 3 months and 36% of them were between 30-40 years.

43% of our participants take alcoholic beverages. 11% smoke cigarette. Only 38% of our participants knew their H.I.V status.

## Conclusion

MSM have good knowledge of condom use but behavioral change is low. These findings show that more HIV counseling/testing centers need to be created for MSM. Lots of MSM need to be reached with intervention to improve knowledge of risky practices associated with high risk men, reduce high-risk sexual practices, reduce barriers to HIV/STI detection and treatment.

